# Calculating an optimal box size for ligand docking and virtual screening against experimental and predicted binding pockets

**DOI:** 10.1186/s13321-015-0067-5

**Published:** 2015-05-15

**Authors:** Wei P. Feinstein, Michal Brylinski

**Affiliations:** Department of Biological Sciences, Louisiana State University, Baton Rouge, LA 70803 USA; Center for Computation & Technology, Louisiana State University, Baton Rouge, LA 70803 USA

**Keywords:** Molecular docking, AutoDock Vina, Docking protocols, Ligand binding site prediction, Ligand virtual screening, Docking box size, Search space

## Abstract

**Background:**

Computational approaches have emerged as an instrumental methodology in modern research. For example, virtual screening by molecular docking is routinely used in computer-aided drug discovery. One of the critical parameters for ligand docking is the size of a search space used to identify low-energy binding poses of drug candidates. Currently available docking packages often come with a default protocol for calculating the box size, however, many of these procedures have not been systematically evaluated.

**Methods:**

In this study, we investigate how the docking accuracy of AutoDock Vina is affected by the selection of a search space. We propose a new procedure for calculating the optimal docking box size that maximizes the accuracy of binding pose prediction against a non-redundant and representative dataset of 3,659 protein-ligand complexes selected from the Protein Data Bank. Subsequently, we use the Directory of Useful Decoys, Enhanced to demonstrate that the optimized docking box size also yields an improved ranking in virtual screening. Binding pockets in both datasets are derived from the experimental complex structures and, additionally, predicted by *e*FindSite.

**Results:**

A systematic analysis of ligand binding poses generated by AutoDock Vina shows that the highest accuracy is achieved when the dimensions of the search space are 2.9 times larger than the radius of gyration of a docking compound. Subsequent virtual screening benchmarks demonstrate that this optimized docking box size also improves compound ranking. For instance, using predicted ligand binding sites, the average enrichment factor calculated for the top 1 % (10 %) of the screening library is 8.20 (3.28) for the optimized protocol, compared to 7.67 (3.19) for the default procedure. Depending on the evaluation metric, the optimal docking box size gives better ranking in virtual screening for about two-thirds of target proteins.

**Conclusions:**

This fully automated procedure can be used to optimize docking protocols in order to improve the ranking accuracy in production virtual screening simulations. Importantly, the optimized search space systematically yields better results than the default method not only for experimental pockets, but also for those predicted from protein structures. A script for calculating the optimal docking box size is freely available at www.brylinski.org/content/docking-box-size.

**Graphical Abstract:**
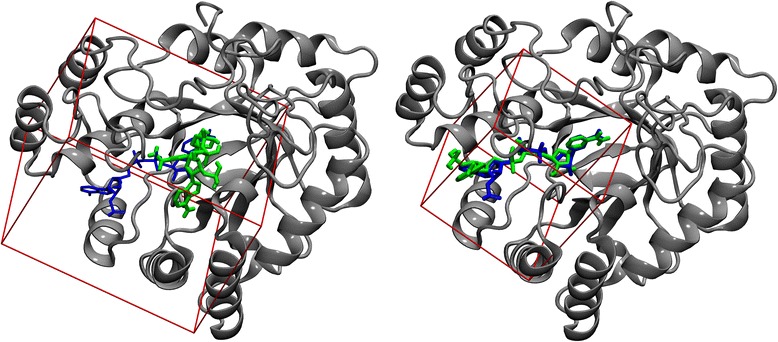
We developed a procedure to optimize the box size in molecular docking calculations. Left panel shows the predicted binding pose of NADP (green sticks) compared to the experimental complex structure of human aldose reductase (blue sticks) using a default protocol. Right panel shows the docking accuracy using an optimized box size.

## Background

Due to advances in information technology, computational approaches have become an important component of modern biological research. Consequently, the past couple of decades have seen a vigorous development of *ad rem* bio-algorithms. For example, protein tertiary structures can be reliably modeled using amino acid sequences [1–3] to help infer their molecular functions [4–6]. Furthermore, putative ligand binding pockets can be confidently predicted from these computer-generated protein models [7–9] and used as target sites for the discovery of new pharmaceuticals [10–12]. Among various technologies developed to date, molecular docking has profound applications in drug design, e.g. it can be used to help identify novel lead compounds [13–15] as well as to support drug repositioning [16–18]. One of the most important techniques in computer-aided drug development is virtual screening, which performs a systematic docking of a large number of drug candidates into target proteins to detect those molecules having a high binding affinity. This procedure reduces the huge initial repository of chemical compounds to a manageable size allowing experimental efforts to focus on the synthesis of a handful of molecules and their subsequent screening against biological targets. In addition to virtual screening supporting the early-stage identification of lead compounds, inverse virtual screening is another cost-reduction strategy, in which a single drug is evaluated against many proteins in order to identify its putative off-targets [19, 20]. On that account, molecular docking holds a great promise to speed up drug discovery, thus it is widely used as an integral part of many currently ongoing drug development projects.

The goal of molecular docking is to predict non-covalent interactions between a ligand and its receptor protein [21, 22]. A typical docking procedure incorporates two important components: a binding pose prediction and the estimation of binding affinity. It is important to note that when ligands bind to their receptor proteins, both molecules may undergo conformational changes, however, allowing for molecular flexibility in docking is computationally challenging because of a large number of rotatable bonds, or the degrees of freedom. Therefore, various methods to sample the conformational space have been developed. For example, systematic sampling techniques [23], Monte Carlo methods [24], genetic search algorithms [25], fragment-based incremental extension methods [26], and rotamer library-based docking using pre-computed low-energy conformations [27] are among many sampling techniques designed to tackle the complexity caused by many degrees of freedom. Each predicted binding pose is assigned a binding affinity that can be calculated using a variety of scoring functions. The most commonly used functions fall into three categories, those employing molecular mechanics force fields such as CHARMM [28] and GROMACS [29], empirical methods implemented in Glide [30] and AutoDock [31], and knowledge-based potentials, e.g. DrugScore [32] and its successor, DSX [33]. As a result of molecular docking, conformational poses generated from a large number of trials within a search space are ranked and the top-ranked conformation is selected as a putative ligand-protein complex. A broad interest in compound docking brought about a significant progress in the development of docking algorithms with many tools currently available; for instance, AutoDock [31, 34], GOLD [35], Glide [30], rDOCK [36], Surflex-Dock [37], FlexX [38], FRED [39], and DOCK [40]. Among these, AutoDock Vina (shortly Vina) [34] is one of the most widely used docking packages in structure-based drug discovery. Compared to its predecessors, Vina features optimized sampling algorithms, new scoring functions, and a support for multithreading to achieve not only higher prediction accuracy but also a significantly improved performance [41].

Molecular docking typically requires a user-defined docking search space, which is explored for possible ligand binding conformations. The selection of a good search space, i.e. the docking box, is a non-trivial task. A narrow search space may produce an insufficient number of conformations, whereas a generously large docking space could result in generating too many irrelevant binding poses. Thus, an optimally confined search space is critical for the success of molecular docking. Many current docking protocols offer a default method for estimating the box size. For example, the default box size in Vina is calculated using experimentally solved protein-ligand complex structures. First, an initial docking box is constructed to enclose the bound ligand, and then the box size is increased in random directions to ensure that the minimum length in any dimension is at least 22.5 Å [34]. Similarly, a docking sphere in GOLD has a radius of 15 Å and it is centered at the position of selected ligand atoms, whereas FRED requires the box size to be expanded to 14,000 Å^3^ based on the coordinates of co-crystallized ligands [42]; these default parameters can be changed by a user.

In the same way as the abovementioned examples, most molecular docking packages require co-crystallized ligands as a starting point to compute the docking box size. However, this information is not always known because only ligand-free experimental structures or homology models are available for many pharmacologically important drug targets. This necessitates using predicted ligand binding sites, which are often less accurate than those extracted from ligand-bound structures. Furthermore, using the default box size calculated from a structure complexed with one ligand may not necessarily yield the highest docking accuracy for another compound, especially when they significantly differ in size. To address these issues, we developed a procedure to customize the box size for individual query ligands in order to maximize the accuracy of molecular docking. Specifically, we systematically examined the docking accuracy as a function of the search space dimensions. Furthermore, we found a correlation between the size of the optimal search space and the radius of gyration of a docking ligand. These results can help fully automate large-scale virtual screening calculations by customizing docking protocols on the fly for individual library compounds.

## Results and discussion

### Optimization of docking box size

**Fig. 1:**
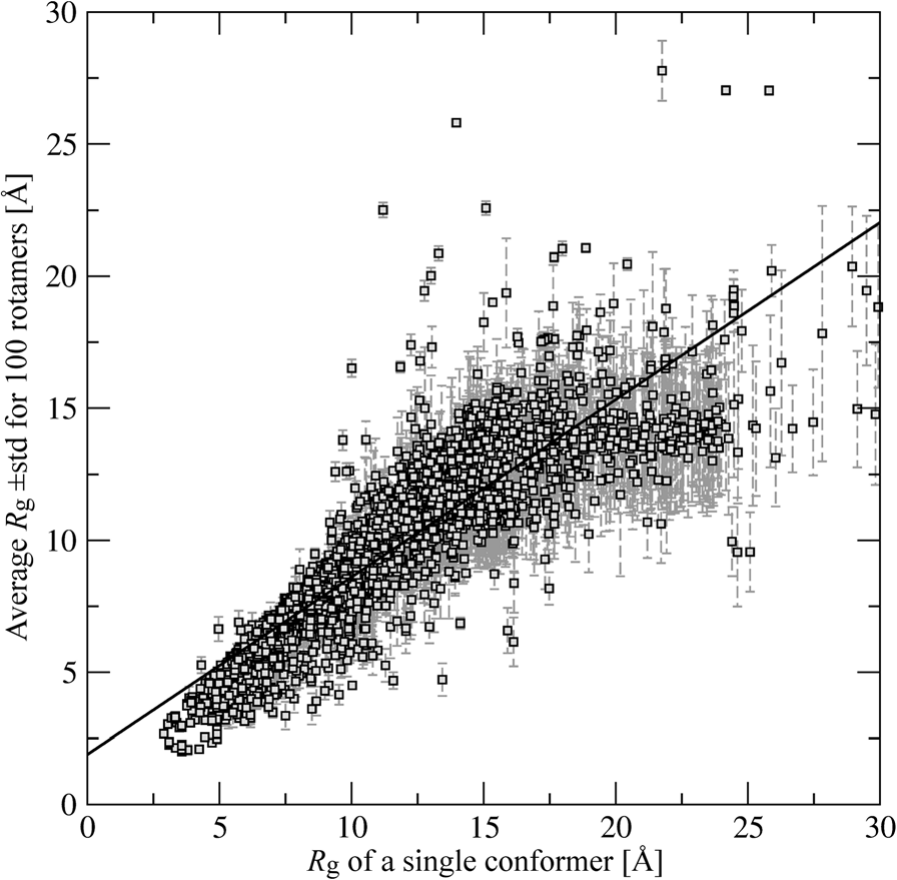
Correlation between the radii of gyration calculated using a single and multiple ligand conformations. For each ligand from the PDB-bench dataset, we calculated the radius of gyration (*R*_g_) for a single low-energy conformation as well as the average *R*_g_±standard deviation for a set of 100 random rotamers. The regression line is shown in black

Molecular docking using Vina is typically conducted using the default box size, which is calculated based on the coordinates of the native ligand interacting with a protein of interest in the experimental structure. However, the coordinates of bound ligands are not always available, in contrast to their chemical structures that are known. Also, the size of a molecule can be effectively described by the radius of gyration, *R*_g_, that is a widely used indicator of the dimensions and the mass distribution of a molecule [43]. For example, a statistical analysis showed a direct relation between *R*_g_ and the compactness of protein structures [44]. In this study, we systematically examine the outcome of molecular docking using different box sizes that depend on the *R*_g_ of query ligands. To maintain a negligible computational overhead, we relate the docking accuracy to *R*_g_ calculated using a single low-energy conformer constructed for each query compound. As shown in Fig. 1, this *R*_g_ value is highly correlated with the average *R*_g_ computed for a set of 100 random rotamers that represent the internal conformational space of individual ligands. The Pearson correlation coefficient is 0.89, therefore, we use *R*_g_ values obtained from single low-energy conformers in the subsequent calculations.

**Fig. 2:**
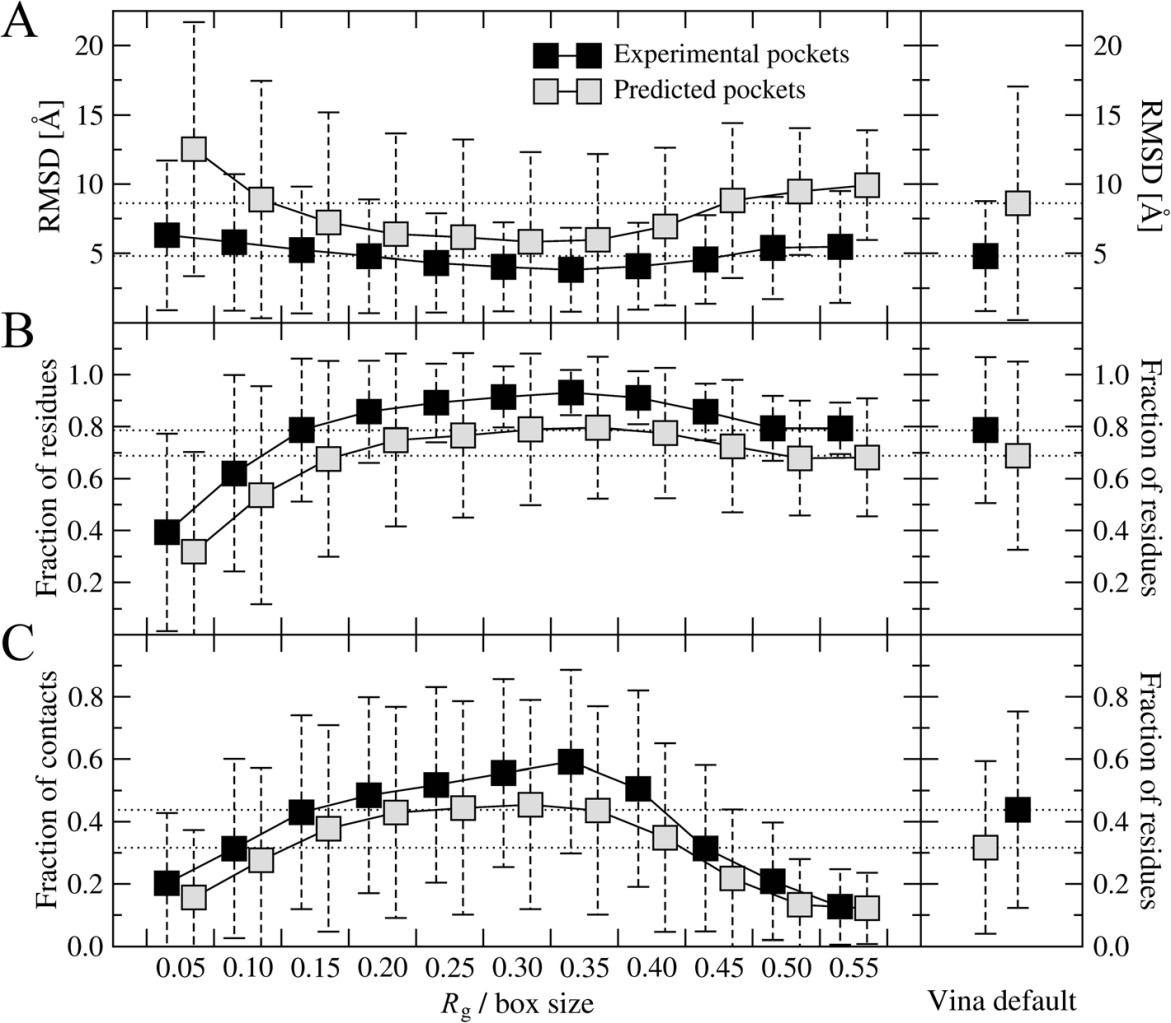
Optimization of the docking box size for Vina using the PDB-bench dataset. Docking accuracy assessed by (**a**) the RMSD over ligand heavy atoms, (**b**) the fraction of recovered binding residues, and (**c**) the fraction of recovered protein-ligand contacts, is plotted as a function of the ratio of the ligand radius of gyration to the box size. The corresponding docking accuracy using the default search space is shown on the right. Squares represent the mean values for each metric and whiskers show the standard deviation. The results obtained for experimental binding sites (black squares) are compared to those predicted by *e*FindSite (gray squares)

**Fig. 3:**
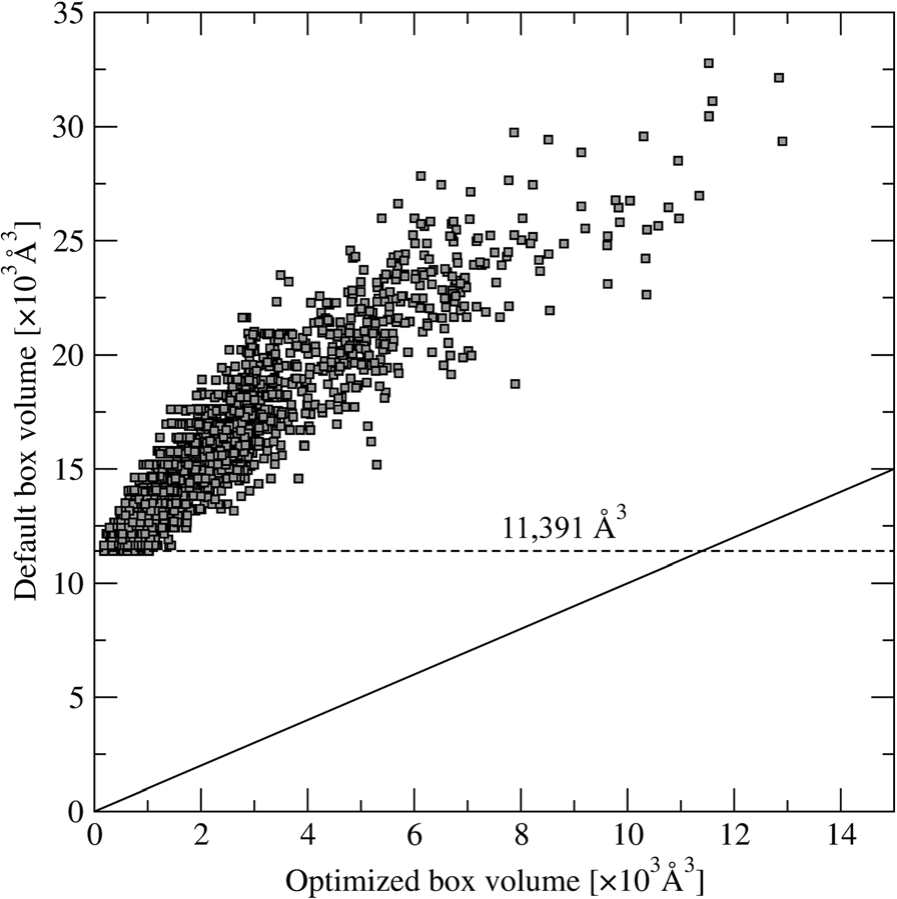
Correlation between default and optimized docking box sizes for the PDB-bench dataset. Each gray square corresponds to one PDB-bench ligand with the default and optimized box sizes represented by their volumes. The solid line is the diagonal and the dashed line shows the minimum volume for a default box calculated as 22.5 Å × 22.5 Å × 22.5 Å

The PDB-bench dataset is used to optimize the box size in order to maximize the docking accuracy of Vina. Specifically, for each target protein, we performed ligand docking using a cubic box centered at the binding site. Edge lengths determining the box size were assigned a value in the rage of 2–36 Å with an incremental interval of 2 Å. To account for ligands that differ in size, we define a relative docking box size as the ratio of the ligand radius of gyration to the actual box size. Figure 2 shows the docking accuracy as a function of the relative box size, assessed by the root-mean-square deviation (RMSD) from the crystal structure calculated over ligand heavy atoms, the fraction of recovered binding residues (non-specific contacts), and the fraction of recovered protein-ligand contacts (specific contacts). Low RMSD values and the high fractions of contacts indicate better ligand binding pose predictions. Regardless of the evaluation metric used, Vina consistently gives the highest prediction accuracy at the *R*_g_ to box size ratio of 0.35, which corresponds to the box size of 2.857 × *R*_g_. Using experimental binding pockets, the optimized box size yields an average RMSD (Fig. 2a), the fraction of binding residues (Fig. 2b) and the fraction of specific contacts (Fig. 2c) of 4.0 Å, 0.92 and 0.58, respectively, whereas the corresponding values for docking calculations using the default box size are 4.9 Å, 0.78 and 0.44 (see the right panel in Fig. 2). Note that the default protocol produces results that are comparable to those reported in other large-scale docking evaluation studies [45]. This improved performance of Vina holds for binding sites predicted by *e*FindSite as well, where using the optimized docking protocol improves RMSD by 2.5 Å and increases the fraction of binding residues and specific contacts by 10 % on average. Figure 3 shows that the optimized box size is systematically smaller than the default one. Therefore, extending the box to at least 22.5 Å in any dimension according to the default procedure may result in scoring failures. On the other hand, box sizes that are too small would likely cause the correct binding mode to extend outside the docking region leading to frequent sampling failures. We note that the optimal box size is calculated directly from the *R*_g_ of a docking ligand, thus it can be obtained for an arbitrary compound. Furthermore, a high prediction accuracy against pockets identified by *e*FindSite demonstrates that Vina can be used in large-scale docking applications, for example, those employing structural genomics targets with computationally detected binding sites.

### Virtual screening benchmarks using DUD-E dataset

Thus far, we established a protocol for calculating the optimal box size for molecular docking that gives the best accuracy in binding pose prediction. Next, we use the Directory of Useful Decoys, Enhanced (DUD-E) to evaluate the performance of Vina in virtual screening. The DUD-E dataset comprises 102 receptor proteins representing many important drug targets, each including sets of bioactive and decoy compounds. Decoy molecules are selected to match the physicochemical properties of the corresponding bioactives, yet they have different topologies. Therefore, DUD-E provides an excellent dataset for benchmarking docking algorithms and scoring functions to help objectively evaluate the capability to differentiate between active and decoy compounds, which is critical for a reliable compound ranking in virtual screening.

First, we docked all molecules to their target proteins using the default and optimized protocols and collected binding affinities reported by Vina. Although the optimized box size was determined individually for each molecule, these calculations produce a negligible overhead since Vina computes grid maps quickly and automatically without storing any intermediate data on the disk [34]. Table 1 shows that on average, actives have higher predicted affinities than decoy compounds (the lower the score, the higher the affinity). For instance, using the default box size gives the absolute difference between the average scores for active and decoy compounds of 0.85 for experimental and 0.73 for predicted binding pockets. When the optimized docking protocol is used in Vina, the differences increase to 0.97 and 0.85, respectively. Table 1 also includes the corresponding *p*-values calculated using the Mann–Whitney *U* test, a nonparametric alternative to the *t*-test [46]. In both cases, *p*-values for the optimized box size are lower than those obtained using the default protocol, suggesting that ligand docking with the optimized box size should more effectively distinguish active compounds from decoys.

**Table 1:** Binding affinity prediction by Vina for the DUD-E dataset. Experimental and predicted binding sites are used in molecular docking with the default and optimized box sizes. Average values and the corresponding standard deviations are reported separately for active and decoy compounds; *p*-values are calculated using the Mann–Whitney *U* test

Class	Experimental binding sites		Predicted binding sites	
	Default	Optimized	Default	Optimized
Actives	−8.70 ± 2.17	−8.25 ± 2.72	−9.00 ± 1.54	−8.48 ± 2.07
Decoys	−7.85 ± 2.07	−7.28 ± 2.53	−8.23 ± 1.28	−7.63 ± 1.91
Difference^a^	0.85	0.97	0.73	0.85
*p*-value	0.139	0.025	0.181	0.043

^a^Absolute value for a difference between the mean binding affinities predicted for actives and decoys

Next, we assess the ranking accuracy in virtual screening using several performance metrics widely used in cheminformatics. These include enrichment factors calculated for the top 1 and 10 % of the ranked library (EF^1 %^ and EF^10 %^), the Boltzmann-Enhanced Discrimination of Receiver Operating Characteristics (BEDROC20) score, the area under the enrichment curve (AUC), and the top fraction of the ranked library that contains 50 % of the active compounds (ACT-50 %). We note that binding sites were identified by *e*FindSite for 77 DUD-E proteins, therefore in addition to the complete DUD-E dataset (D101, experimental binding sites only), we report the results for this subset of 77 proteins (D77, experimental and predicted pockets). Table 2 shows that when the default protocol is used, the average EF^1 %^, EF^10 %^, BEDROC20, AUC and ACT-50 % for the D77 are 8.126, 3.324, 0.229, 0.697 and 0.244, respectively. Using the optimized box size in Vina improves the performance of virtual screening to 8.131, 3.443, 0.234, 0.703 and 0.234. As expected, when virtual screening is carried out for predicted pockets, the overall accuracy is somewhat lower than that for experimental binding sites. Nevertheless, the optimized docking protocol systematically improves the ranking capabilities of Vina; for instance, EF^1 %^ increases from 7.670 to 8.205, EF^10 %^ increases from 3.193 to 3.283 and BEDROC20 increases from 0.218 to 0.229. Although not all differences are statistically significant as evaluated by the Wilcoxon signed-rank test [47] (e.g. these calculated for EF^1 %^), many *p*-values reported in Table 2 are either within (e.g. EF^10 %^ for experimental pockets in D101 and BEDROC20 for predicted pockets) or slightly above (e.g. BEDROC20 for experimental pockets in D101, and AUC for experimental and predicted pockets in D77) the significance level of 0.05.

**Table 2:** Accuracy of virtual screening by Vina for the DUD-E dataset. Experimental and predicted binding sites are used in molecular docking with the default and optimized box sizes. Average values and the corresponding standard deviations are reported; *p*-values are calculated using the Wilcoxon signed-rank test

Metric	Dataset^a^	Experimental binding sites			Predicted binding sites		
		Default	Optimized	*p*-value	Default	Optimized	*p*-value
EF^1 %^	D77	8.126 ± 7.881	8.131 ± 7.567	0.795	7.670 ± 7.886	8.205 ± 8.245	0.108
	D101	7.714 ± 8.311	7.782 ± 7.806	1.000	-	-	-
EF^10 %^	D77	3.324 ± 1.738	3.443 ± 1.827	0.142	3.193 ± 1.701	3.283 ± 1.754	0.073
	D101	3.137 ± 1.717	3.295 ± 1.815	0.034	-	-	-
BEDROC20	D77	0.229 ± 0.140	0.234 ± 0.141	0.274	0.218 ± 0.136	0.229 ± 0.142	0.041
	D101	0.214 ± 0.138	0.223 ± 0.140	0.067	-	-	-
AUC	D77	0.697 ± 0.130	0.703 ± 0.123	0.065	0.688 ± 0.130	0.689 ± 0.131	0.082
	D101	0.690 ± 0.125	0.697 ± 0.118	0.006	-	-	-
ACT-50 %	D77	0.244 ± 0.165	0.234 ± 0.151	0.030	0.251 ± 0.160	0.251 ± 0.163	0.101
	D101	0.254 ± 0.159	0.240 ± 0.146	0.006	-	-	-

^a^D77 is the subset of DUD-E containing only those proteins whose binding sites are detected by *e*FindSite, D101 is the entire DUD-E dataset

**Fig. 4:**
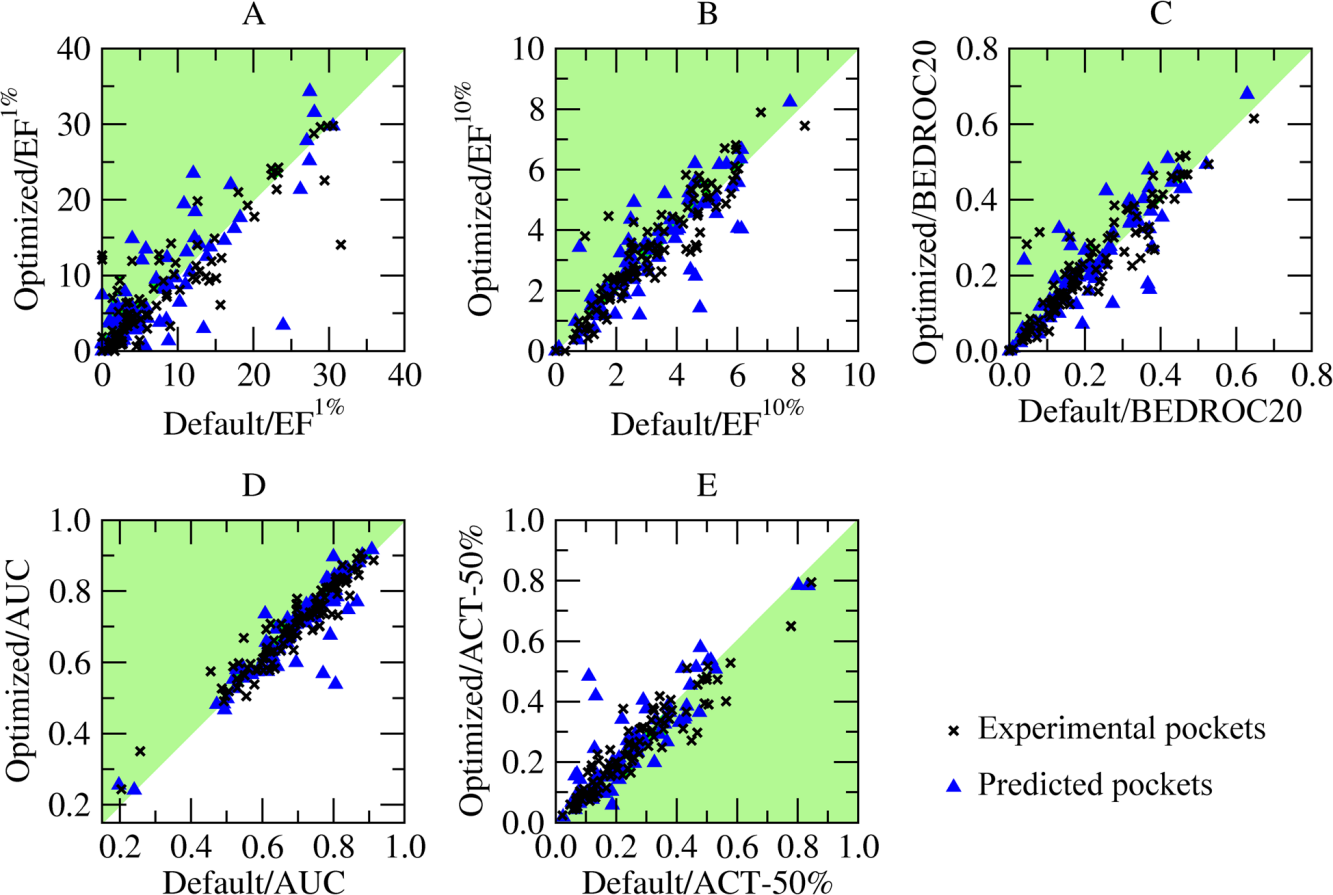
Virtual screening benchmarks of Vina against the DUD-E dataset. Ranking accuracy using the default and optimized box size is evaluated by the enrichment factor for the top (**a**) 1 % and (**b**) 10 % of the ranked library, (**c**) Boltzmann-Enhanced Discrimination of Receiver Operating Characteristics, (**d**) the area under the enrichment curve, and (**e**) the top fraction of the ranked library that contains 50 % of actives. The results obtained for experimental pockets (black crosses) are compared to binding sites predicted by *e*FindSite (blue triangles). Green areas highlight those target proteins for which the optimized box size yields better results than the default protocol

Figure 4 presents the results obtained for individual proteins in the D77 dataset with the green areas highlighting those targets for which the optimized box size yields a higher ranking accuracy than the default protocol. Using EF^1 %^, EF^10 %^, BEDROC20, AUC and ACT-50 % as the evaluation metric, the optimized box size improves compound ranking for experimental binding sites (black crosses in Fig. 4) in 60, 66, 57, 61 and 65 % of the cases, respectively. The optimized protocol yields slightly higher improvements than the default procedure for computationally predicted pockets (blue triangles in Fig. 4), where better ranking is obtained for 66, 64, 64, 60 and 65 % of the target proteins. This analysis demonstrates that the accuracy of virtual screening can be quantitatively improved for about two-thirds of the cases by simply adjusting the docking box size based on the *R*_g_ of screening compounds.

### A case study for ligand binding pose prediction

**Fig. 5:**
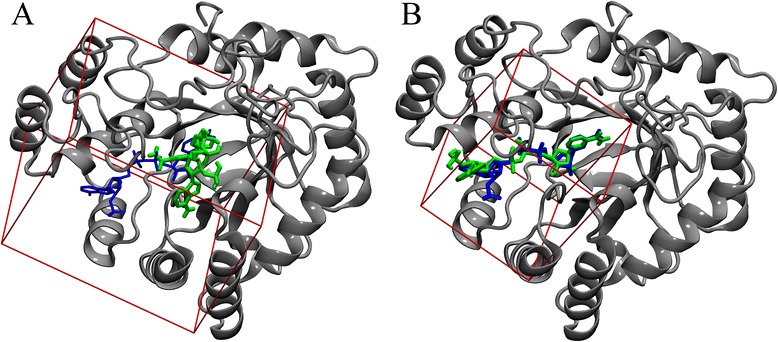
Case study for molecular docking by Vina. Gray ribbons represent human aldose reductase with (**a**) the default and (**b**) the optimized docking boxes shown in red. Predicted binding poses for NADP (green sticks) are compared to that in the experimental complex structure (blue sticks)

To illustrate the improvement in docking accuracy using the optimized box size, we selected a 315 aa human aldose reductase holoenzyme complexed with nicotinamide-adenine-dinucleotide phosphate, NADP (PDB-ID: 1ads, chain A); this enzyme has been implicated in the development of diabetic and galactosemic complications [48]. Figure 5 shows the search space for ligand docking by Vina and the corresponding predicted binding poses. The default box size of *x* = 30.66 Å, *y* = 27.98 Å and *z* = 22.50 Å (Fig. 5a) was calculated based on the conformation of NADP bound to its target in the crystal structure, whereas the optimized box size of *x* = *y* = *z* = 18.88 Å (Fig. 5b) was calculated from the *R*_g_ of NADP. The predicted binding poses of NADP (green sticks) are compared to the ligand orientation in the complex crystal structure (blue sticks). The default protocol generated a large docking box and produced the binding pose with an RMSD from native of 13.9 Å. In contrast, a smaller box was constructed by the optimized method, which resulted in the final conformation of NADP that has an RMSD of 2.7 Å. Thus, using the optimized search space significantly improved the accuracy of NADP binding pose prediction by Vina.

## Conclusions

Molecular docking has profound applications in drug discovery and development. Selecting an appropriate search space is critical to achieve high prediction accuracy in structure-based virtual screening. Here, we developed a procedure to customize the box size for individual query ligands in order to maximize the docking accuracy. Furthermore, we found a correlation between the dimensions of the optimal search space and the radius of gyration of a docking compound. This docking protocol essentially brings down the number of scoring failures resulting from too generous box sizes, simultaneously avoiding sampling failures caused by a search space that is too narrow. Large-scale benchmarking calculations on the DUD-E dataset show that using the optimized box size also improves the ranking accuracy in virtual screening over the default protocol. Importantly, the enhanced docking performance is also validated in simulations against predicted binding sites, which expands the scope of molecular docking by including computationally detected pockets. In summary, these results can help fully automate large-scale virtual screening calculations by customizing docking protocols on the fly for individual library compounds. A script for calculating the optimal docking box size is freely available at www.brylinski.org/content/docking-box-size.

## Methods

### Molecular docking using autodock vina

AutoDock Vina (version 1.1.2) [34] is used in this project to conduct molecular docking. Target protein structures are converted to the required PDBQT format using MGL Tools (version 1.5.4) [31]. Open Babel (version 2.3.1) [49] is used to add polar hydrogens and partial charges to ligand atoms as well as to convert these molecules to the PDBQT format. The default box size is calculated following the protocol outlined by the authors of Vina [34]. Briefly, an initial docking box is calculated from the coordinates of a bound ligand in the crystal structure, and the box dimensions in *x*, *y* and *z* are increased by 10 Å. Additionally, one of the two directions in each dimension is randomly chosen and further increased by 5 Å. Finally, if the box size in any dimension is smaller than 22.5 Å, it is extended to this value. In this study, an experimental binding site is defined as the geometric center of a ligand bound to the target protein, whereas the computationally predicted binding pocket center is obtained from *e*FindSite [9]. Docking simulations using predicted pockets start with a random ligand conformer generated by obconformer from Open Babel [49]; moreover, the ligand is randomly spun around all axes in order to avoid providing the docking program with any structural information on the native binding pose. All ligands are also translated so that their geometric centers overlap with predicted pocket centers.

### Protein data bank benchmark dataset

The benchmarking dataset, referred to as the PDB-bench, is used to optimize box sizes in order to yield the highest docking accuracy. PDB-bench was compiled from the Protein Small Molecule Database [50] and the Protein Data Bank (PDB) [51] by including only proteins 50–600 residues in length with the redundancy removed at 40 % pairwise sequence identity using PISCES [52]. The length constraints are imposed due to the subsequent use of protein threading, however, these do not exclude pharmacologically relevant molecules such as G-protein coupled receptors (GPCRs) and protein kinases. Furthermore, we selected those proteins for which at least three weakly homologous and structurally related ligand-bound templates were detected by meta-threading using *e*Thread [3]. We note that weak homology is defined by the maximum sequence identity of 40 %, and the structural similarity of ≥0.4 TM-score [53] as reported by Fr-TM-align [54]. Furthermore, only non-covalently bound small organic compounds with 6–100 heavy atoms were selected. As the result, a representative and non-redundant PDB-bench comprises 3,659 experimental structures of protein-ligand complexes; this dataset is available at www.brylinski.org/content/docking-box-size.

### Optimal box size and ligand radius of gyration

In order to optimize the search space, we perform a series of docking calculations for each target using a cubic box whose edge lengths range from 2 to 36 Å with a small incremental step size of 2 Å to ensure a fine-grained sampling. Next, we analyze docking accuracy as a function of the size of a query compound size by calculating the ratio of the radius of gyration of a ligand (*R*_g_) to the box size. *R*_g_ is defined as follows:

1 $$ {R}_g = \sqrt{\frac{1}{N}{\displaystyle {\sum}_{k=1}^N\left|{\overset{\rightharpoonup }{r}}_k-{\overset{\rightharpoonup }{r}}_{center}\ \right|{}^2\ }\kern0.5em } $$

where *N* is the total number of ligand heavy atoms, the vector ${\overset{\rightharpoonup }{r}}_k$ corresponds to the Cartesian coordinates of each heavy atom, and ${\overset{\rightharpoonup }{r}}_{center}$ represents the geometric center of a ligand.

By default, we calculate *R*_g_ for a single low-energy conformer generated for each query compound by obconformer from Open Babel [49]. For comparison, we also calculated the average values of *R*_g_ ± standard deviation using sets of 100 random rotamers generated by obrotamer (Open Babel [49]) for PDB-bench ligands.

### Directory of useful decoys, enhanced dataset

DUD-E, an enhanced version of the DUD dataset [55], comprises a diverse set of 101 proteins including many pharmacologically important targets such as ion channels and GPCRs [56]. DUD-E features 22,886 experimentally validated active compounds with an average number of 224 ligands per each protein target, and over 1,000,000 decoy molecules at an approximate ratio of 50 per 1 active compound. These decoys have similar chemical properties yet different topologies than the corresponding active compounds. Therefore, the DUD-E dataset allows performing rigorous and unbiased tests of docking algorithms, scoring functions and virtual screening tools [57, 58]. Similar to the PDB-bench dataset, we carried out docking calculations using experimental pocket centers calculated from 101 representative complex structures included in DUD-E (the D101 set). Furthermore, we evaluate the accuracy of virtual screening for a subset of 77 proteins whose binding sites were successfully predicted by *e*FindSite (the D77 set). A binding site prediction is considered successful when the distance between the predicted and experimental pocket center is below 8 Å.

### Evaluation metrics for molecular docking and virtual screening

Docking accuracy is assessed by the root-mean-square deviation (RMSD) from the crystal structure calculated over ligand heavy atoms [59], and the fraction of recovered protein-ligand contacts. Specific interatomic contacts between ligand and protein heavy atoms are identified using the LPC program [60]. In addition, we use the fraction of non-specific contacts between ligand heavy atoms and protein residues, where all atoms belonging to the same residue are equivalent. More accurate docking predictions are characterized by lower RMSD values as well as higher fractions of specific and non-specific contacts compared to those less accurate.

Virtual screening results are assessed by several commonly used evaluation metrics. Enrichment factors EF^1 %^ and EF^10 %^ count the fraction of actives in the top 1 and 10 % of the ranked library, respectively. In order to address the “early recognition problem”, we use the Boltzmann-Enhanced Discrimination of Receiver Operating Characteristics (BEDROC20) score that calculates 80 % of the enrichment from the top 8 % of the ranked library [61]. In addition, we evaluate the area under the enrichment curve (AUC) that determines the discriminative capability by measuring the distribution of actives over the entire library. Finally, we calculate ACT-50 %, which corresponds to the top fraction of the ranked library that contains half of the active compounds.

## Abbreviations

ACT-50 %: The fraction of the ranked library that contain 50 % actives
AUC: The area under the enrichment curve
BEDROC20: Boltzmann-Enhanced Discrimination of Receiver Operating Characteristics
DUD-E: Directory of Useful Decoys, Enhanced
EF^1 %^: The enrichment factor for the top 1 % of the ranked library
EF^10 %^: The enrichment factor for the top 10 % of the ranked library
GPCRs: G-protein coupled receptors
NADP: Nicotinamide adenine dinucleotide phosphate
PDB: Protein Data Bank
*R*_g_: The radius of gyration
RMSD: The root-mean-square deviation
Vina: AutoDock Vina
